# COVID-19 transmission in group living environments and households

**DOI:** 10.1038/s41598-021-91220-4

**Published:** 2021-06-02

**Authors:** Tetsuya Akaishi, Shigeki Kushimoto, Yukio Katori, Shigeo Kure, Kaoru Igarashi, Shin Takayama, Michiaki Abe, Junichi Tanaka, Akiko Kikuchi, Ko Onodera, Tadashi Ishii

**Affiliations:** 1 Department of Education and Support for Regional Medicine, Tohoku University Hospital, Seiryo-machi 1-1, Aoba-ku, Sendai, Miyagi 980-8574 Japan; 2 Tohoku University Outpatient Clinic for COVID-19 Testing, Tohoku University, Sendai, Japan; 3 Division of Emergency and Critical Care Medicine, Tohoku University Graduate School of Medicine, Sendai, Miyagi Japan; 4 Department of Otolaryngology-Head and Neck Surgery, Tohoku University Graduate School of Medicine, Sendai, Miyagi Japan; 5 Department of Pediatrics, Tohoku University Graduate School of Medicine, Sendai, Miyagi Japan; 6 Division of Craniofacial Anomalies, Tohoku University Graduate School of Dentistry, Sendai, Miyagi Japan; 7 Department of General Practitioner Development, Tohoku University Graduate School of Medicine, Sendai, Japan

**Keywords:** Infectious diseases, Viral infection

## Abstract

The coronavirus disease 2019 (COVID-19) pandemic caused by severe acute respiratory syndrome coronavirus 2 (SARS-CoV-2) is currently the world’s largest public health concern. This study evaluated COVID-19 transmission risks in people in group living environments. A total of 4550 individuals with a history of recent contact with patients at different places (dormitory/home/outside the residences) and levels (close/lower-risk) were tested for SARS-CoV-2 viral RNA using a nasopharyngeal swab test between July 2020 and May 2021. The test-positive rate was highest in individuals who had contact in dormitories (27.5%), but the rates were largely different between dormitories with different infrastructural or lifestyle features and infection control measures among residents. With appropriate infection control measures, the secondary transmission risk in dormitories was adequately suppressed. The household transmission rate (12.6%) was as high as that of close contact outside the residences (11.3%) and accounted for > 60% of the current rate of COVID-19 transmission among non-adults. Household transmission rates synchronized to local epidemics with changed local capacity of quarantining infectious patients. In conclusion, a group living environment is a significant risk factor of secondary transmission. Appropriate infection control measures and quarantine of infectious residents will decrease the risk of secondary transmission in group living environments.

## Introduction

The world is currently struggling with the outbreak of coronavirus disease 2019 (COVID-19) caused by severe acute respiratory syndrome coronavirus 2 (SARS-CoV-2)^1,2^. Like many other countries, Japan has also experienced irregular waves of nationwide epidemics since 2020^3,4^. To break the chain of COVID-19 transmission, both SARS-CoV-2 screening tests and contact tracing have been indispensable for facilitating public health policies against the pandemic^5^ and have been applied as a national policy in the country^6–8^. Recent contact history with COVID-19 patients is an established powerful predictor of positive SARS-CoV-2 test results^9–11^. To date, governments in many countries have established a definition for close contact with COVID-19 patients, focusing on risk behaviors that may predispose droplet infections^12^, and have implemented quarantine measures (e.g., hospitals, quarantine hotels) for infectious patients and high-risk individuals^13^. It has also been suggested that the SARS-CoV-2 transmission rate may be affected by multiple factors and might differ between locations (e.g., school, workplace, home, or eating place) and behaviors (e.g., speaking, sharing foods, physical contact in sports activity, kissing) of the contact episode^14,15^. Furthermore, the occurrence of clusters at community events or in group living facilities is also suggested to play a significant role in the spread of the infection^6^. However, the exact secondary COVID-19 transmission risk among people living in group living environments, such as dormitories, or the risk factors that may increase the secondary transmission rate among the residents of such environments, have not yet been fully examined.

In response to request from the local government (Miyagi prefecture and Sendai City) to test a large number of people, we established a drive-through COVID-19 testing center in April, 2020, to perform nasopharyngeal swab testing for the subsequent SARS-CoV-2 real-time reverse transcription polymerase chain reaction (RT-PCR) assay^16^. We started to routinely collect the detailed data regarding the history of contact with COVID-19 patients from the tested individuals since July, 2020. In this study, we analyzed the SARS-CoV-2 RT-PCR test results stratified by the location and level of contact with patients with COVID-19 to estimate the risk of each potential contact-related predisposing factor (contact in group living facility, household contact, close contact outside the residence) in the transmission of COVID-19.

## Results

### SARS-CoV-2 test positivity rate by the place and level of contact

**Figure 1:**
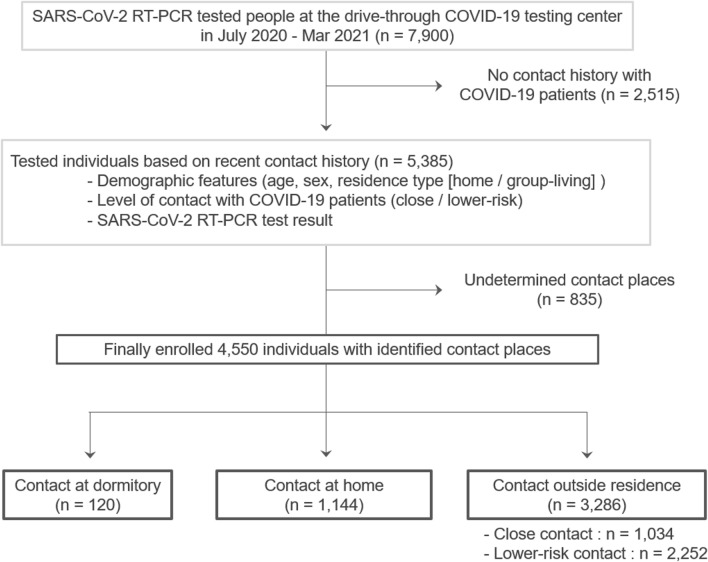
Study design flowchart with three groups at different contact places. The flowchart illustrates the inclusion and exclusion processes of the study and the categorization into three groups according to the different places of contact (dormitory, home, and outside residence) with the COVID-19 patients. COVID-19, coronavirus disease; RT-PCR, reverse transcription polymerase chain reaction; SARS-CoV-2, severe acute respiratory syndrome coronavirus 2.

**Figure 2:**
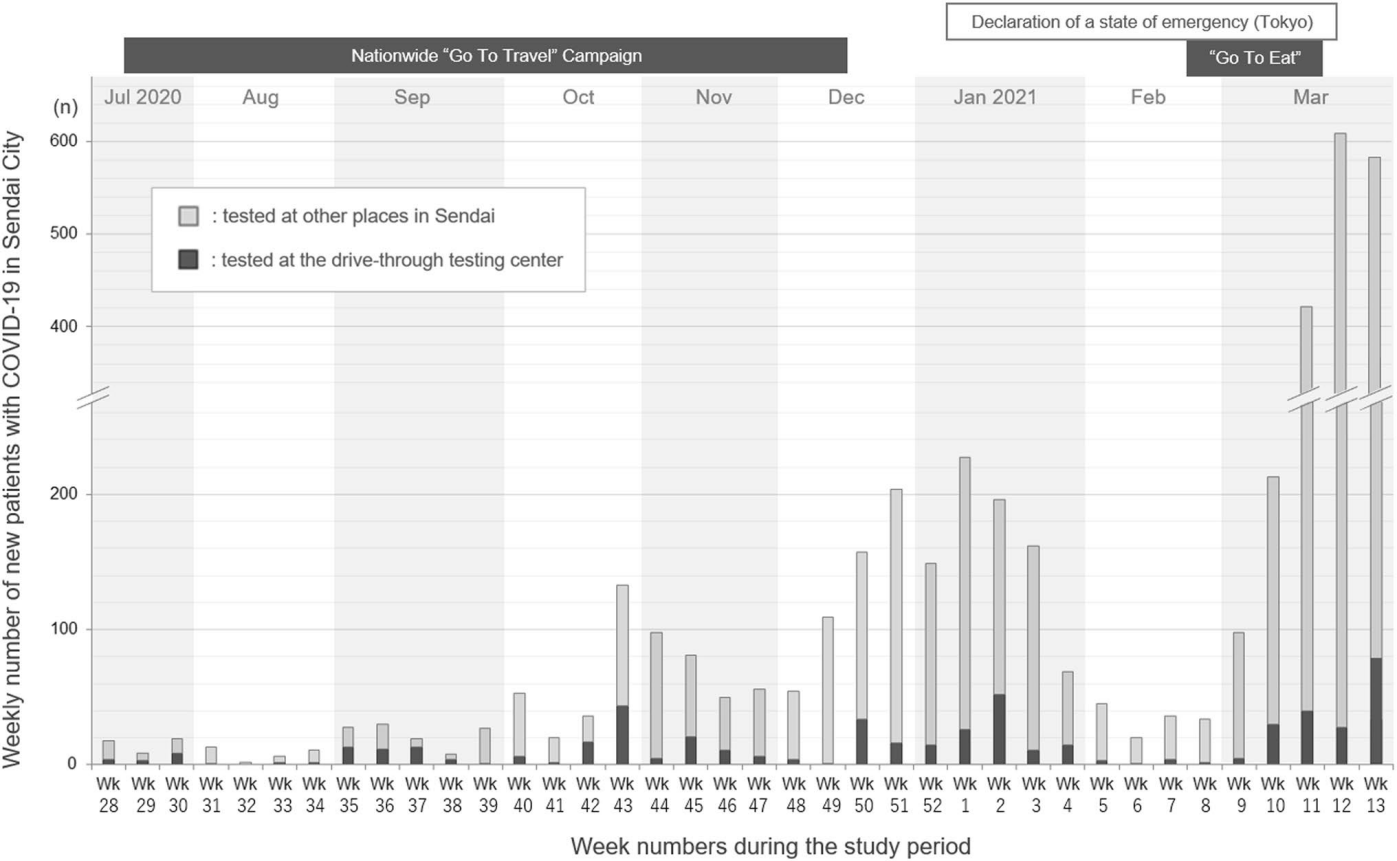
Weekly number of new patients with COVID-19 in Sendai City from July 2020 to March 2021. The black bars illustrate the weekly number of patients newly diagnosed with COVID-19 tested at a drive-through COVID-19 testing center and were initially recruited for this study. The gray bars above the black bars represent the weekly number of patients newly diagnosed with COVID-19 tested at other testing facilities in Sendai City who were not enrolled in this study. The local government announced the daily numbers of new local patients. COVID-19, coronavirus disease; RT-PCR, reverse transcription polymerase chain reaction.

Our study design is shown in Fig. 1. During the study period between July 2020 and March 2021, a total of 7900 individuals were tested for SARS-CoV-2 viral RNA by RT-PCR nasopharyngeal swab test at our testing center. This population included 497 SARS-CoV-2 swab test-positive patients, accounting for 12.8% of all confirmed COVID-19 patients in Sendai City during the same period. The daily number of new COVID-19 patients in Sendai City and the number of those tested at our testing center during the study period are shown in Fig. 2.

Among the whole population tested at the drive-through testing center, 2515 individuals had no recent history of contact with COVID-19 patients and were therefore excluded from subsequent analyses. Among the remaining 5385 individuals with a history of recent contact with patients, 4550 identified their place of contact (e.g., home, dormitory, school, workplace, hotels, restaurants, bars, cars, or other places), and the remaining 835 did not have certain data regarding the place of contact. Among the 4550 participants with reliable data regarding the place of contact, 355 participants (7.8%; 95% confidence interval [CI], 7.1–8.6%) tested positive for SARS-CoV-2 on RT-PCR. Among the 4550 individuals, 2179 (47.9%) had a close contact history and the remaining 2371 (52.1%) had a lower-risk contact. In the first group, 259 (11.9%; 95% CI, 10.6–13.3%) tested positive on RT-PCR. In the second group with a low-risk contact history, 96 (4.0%; 95% CI, 3.3–4.9%) tested positive on RT-PCR. The rate of RT-PCR test positivity was significantly higher in those with a close contact than in those with a lower risk contact (effect size φ = 0.146, chi-square test, *p* < 0.0001).

**Figure 3:**
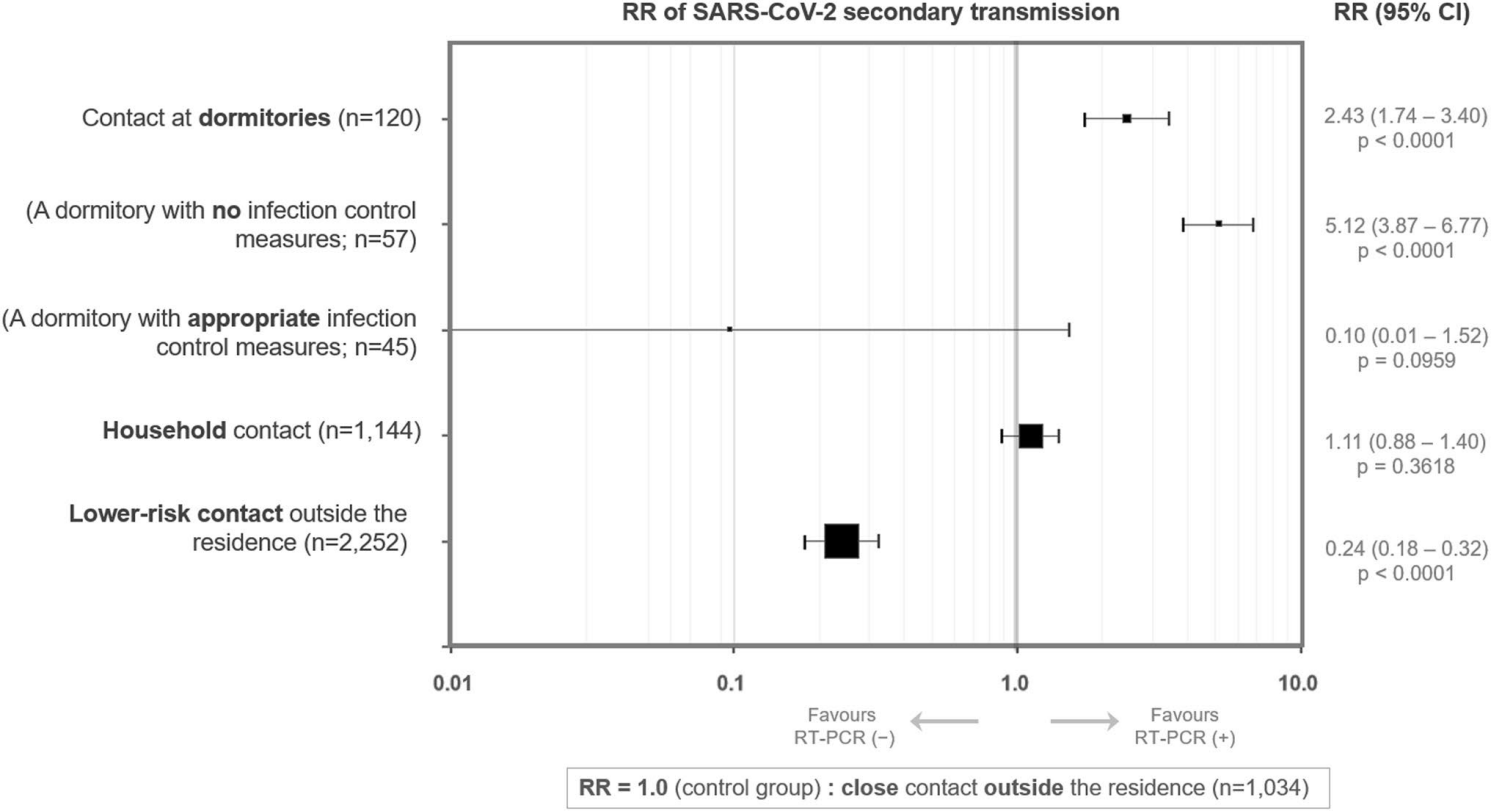
Relative risks of acquiring the COVID-19 infection by place of contact with COVID-19 patients. The figure shows subgroup-specific risk ratios (RR) for subjects in different groups divided by the places and levels of recent contact history with COVID-19 patients. The crude RR for each subgroup is plotted as a black square, and the size of each plot is proportional to the number of subjects in each subgroup. Error bars represent 95% confidence intervals of the RR. Individuals with recent close contact outside their residences (n = 1034) were considered as the control group, corresponding to an RR value of 1.0 (the solid vertical line). The values of RR above 1.0 indicate that people in each group are more likely to have a positive SARS-CoV-2 RT-PCR test result. An RR value of < 1.0 indicates a lower RT-PCR test positivity rate. The calculated RR are plotted on a logarithmic scale. COVID-19, coronavirus disease; RR, risk ratios; RT-PCR, reverse transcription polymerase chain reaction; SARS-CoV-2, severe acute respiratory syndrome coronavirus 2.

The 4550 individuals with an identified place of contact with patients were further divided into the following three groups according to the place of contact: (1) dormitory contact group (n = 120), (2) household contact group (n = 1144), and (3) contact outside the residence group (n = 3286). The last group was further divided into those with a close contact (n = 1034) and those with a lower risk contact (n = 2252). The demographic and clinical features of the enrolled individuals, stratified by place and level of recent contact with patients, are shown in Table 1. The 120 participants who contacted with COVID-19 patients at dormitories consisted of 11 with close contact and 109 with lower-risk contact. The 1144 individuals with household contact consisted of 1134 with close contact and 10 with lower-risk contact. The SARS-CoV-2 test positivity rate was highest in the dormitory contact group (27.5%; 95% CI, 20.3–36.1%), followed by the household contact group (12.6%; 95% CI, 10.8–14.6%), and the close contact outside the residence group (11.3%; 95% CI, 9.5–13.4%). The positivity rate was lowest in the lower-risk contact outside the residence group (2.7%; 95% CI, 2.1–3.5%). The calculated crude risk ratio (RR) and its 95% CI of acquiring COVID-19 infection in each group, considering the 1034 individuals with close contact outside the residences as the control group, is listed in Fig. 3.

**Table 1:** Demographic features of the participants by the place of contact with COVID-19 patients.

	Contact at dormitory	Contact at home	Contact at other places outside residence	
			Close contact	Lower-risk contact
n	120	1144	1034	2252
Male, n (%)*	105 (87.5%)	485 (42.4%)	573 (55.4%)	1168 (51.9%)
Age ^†^	24 (22–27) years	36 (15–54) years	23 (15–44) years	15 (6–28) years
0–17 years, n (%)*	0 (0.0%)	333 (29.1%)	389 (37.6%)	1358 (60.3%)
18–64 years, n (%)*	119 (99.2%)	642 (56.1%)	561 (54.3%)	824 (36.6%)
65 + years, n (%)*	1 (0.8%)	169 (14.8%)	84 (8.1%)	70 (3.1%)
Close contact, n (%)	11 (9.2%)	1134 (99.1%)	1034 (100.0%)	0 (0.0%)
Lower risk contact, n (%)	109 (90.8%)	10 (0.9%)	0 (0.0%)	2252 (100.0%)
SARS-CoV-2 RT-PCR test positivity, n (%)*				
Total	33 (27.5%)	144 (12.6%)	117 (11.3%)	61 (2.7%)
Non-adult (< 18 years old)	-	43/333 (12.9%)	11/389 (2.8%)	12/1358 (0.9%)
Adult (≥ 18 years old)	33/120 (27.5%)	101/811 (12.5%)	106/645 (16.4%)	49/894 (5.5%)

Close contact was defined by the existence of the following four criteria: (1) contact with a patient between 2 days before and 14 days after the onset of symptoms, (2) no usage of masks, (3) distance within 1 m, and (4) more than 15 min of contact. Lower risk contact was defined as presence in the same facility as COVID-19 patients, but without fulfilling the above-described criteria of close contact.

COVID-19, coronavirus disease; n.a., not available; RT-PCR, reverse transcription-polymerase chain reaction; SARS-CoV-2, severe acute respiratory syndrome coronavirus-2.

*Numbers and percentages in the column (i.e., among subjects in each contact level group); ^†^ median and interquartile range (25–75 percentiles).

**Figure 4:**
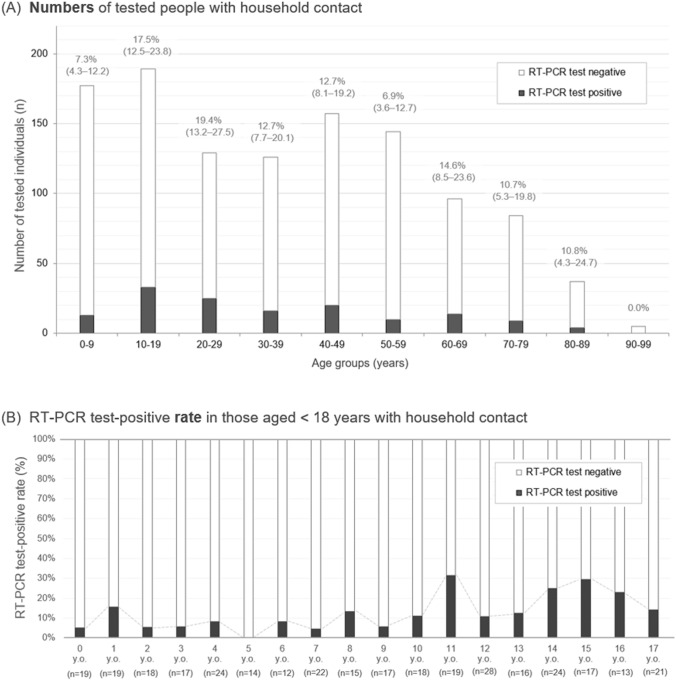
Histogram of tested individuals after household contact with a patient with COVID-19 by age group. The figures present (**A**) a histogram of the number (n) of tested individuals after household contact with a family member with COVID-19 separated into 10-year age intervals among all 1144 enrolled individuals with household contact. (**B**) RT-PCR test-positive rate (%) by age after household contact in 333 non-adults aged < 18 years. Black bars represent the proportion of positive RT-PCR test results, and white bars above the black bars illustrate the proportion of negative RT-PCR test results. The percentage and range shown in panel (**A**) indicate the proportion of positive RT-PCR tests and the 95% confidence interval in each age group, respectively.

As shown in the bottom of the table, when we further divided each group into non-adults (< 18 years old) and adults (≥ 18 years old), the risk of household transmission in non-adults was significantly higher than the risk of close contact outside the residence (mostly at schools) for non-adults (RT-PCR test positivity rate: 12.9% vs. 2.8%, φ = 0.191, *p* < 0.0001). In contrast, the risk of household transmission in adults was slightly lower than their risk of close contact outside the residence (12.5% vs. 16.4%, φ = 0.057, *p* = 0.0307). The household transmission rate after contact with an infected family member stratified by age group is shown in Fig. 4. The household secondary transmission rates were similar in all age groups at 5–20%, but were suggested to be slightly lower in children aged < 10 years than in other groups (7.3% vs. 13.5%, chi-square test, *p* = 0.0222). Among the non-adults, the RT-PCR test-positive rate after household contact appeared to be slightly lower in children aged 0–10 years than in children aged 11–17 years (Fig. 4B).

### Logistic regression analysis for SARS-CoV-2 test positivity

Binary logistic regression analysis was performed to determine the independent risk of contact with patients in group living environments by enrolling 4550 individuals with identified places of contact with patients with COVID-19. SARS-CoV-2 test positivity was used as the response variable. The explanatory variables used were age, sex, close contact history, and contact at a dormitory or household contact with an infected family member. The results of the logistic regression analysis with these two sets of explanatory variables are presented in Table 2. Both close contact and dormitory life without appropriate infection control measures were confirmed to be independent significant risks for acquiring COVID-19 (upper half of the table). Meanwhile, household contact was not confirmed as a significant risk of acquiring COVID-19 independent of the closeness of contact, possibly because almost all individuals with household contact were categorized as having a close contact history (lower half of the table).

**Table 2:** Binary logistic regression analysis for potential predictors of SARS-CoV-2 test-positive results.

	B	SEB	Wald	OR (95%CI)	*p* value
**(When “contact at dormitory” is used as an explanatory variable)**					
(Constant)	− 3.750	0.150	626.912	0.02 (0.02–0.03)	< 0.0001
Age	+ 0.014	0.003	32.638	1.01 (1.01–1.02)	< 0.0001
Sex (Male)	− 0.073	0.116	0.390	0.93 (0.74–1.17)	0.5322
Close contact history	+ 1.247	0.140	79.157	3.48 (2.64–4.58)	< 0.0001
Contact at dormitory	+ 2.342	0.238	96.709	10.40 (6.52–16.59)	< 0.0001
**(When “household contact” is used as an explanatory variable)**					
(Constant)	− 3.521	0.138	656.215	0.03 (0.02–0.04)	< 0.0001
Age	+ 0.014	0.002	31.970	1.01 (1.01–1.02)	< 0.0001
Sex (Male)	+ 0.065	0.113	0.328	1.07 (0.85–1.33)	0.5670
Close contact history	+ 0.955	0.145	43.343	2.60 (1.96–3.45)	< 0.0001
Household contact	+ 0.061	0.135	0.206	1.06 (0.82–1.39)	0.6500

Logistic regression analyses were performed for 4550 individuals with identified places of contact with COVID-19 patients. The upper half shows the results when contact in a dormitory, irrespective of the closeness of contact, is used as an explanatory variable. The lower half shows the results when household contact is used instead. The OR values are equivalent to exp(B). Wald χ^2^ statistics (Wald) were calculated using the formula ${\left(B/SEB\right)}^{2}$, which is a marker of the significance of each coefficient in the predictive model.

B, unstandardized regression coefficient; CI, confidence interval; SEB, standard error of the coefficient; OR, odds ratio.

### Details of the contact in dormitories

As shown above, contact with COVID-19 patients in dormitories was suggested as a significant risk factor for COVID-19 transmission, compatible with the risk of close contact outside the residences. Thus, we further investigated the details of the dormitories in the contact episodes of this study. There were 120 dormitory residents from three dormitories: dormitory A of School #1 (n = 57), dormitory B of School #2 (n = 45), and dormitory C of School #3 (n = 18). Regarding the total participants, the crude RR (95% CI) of living in a dormitory, when considering the other 4430 people as the reference group (i.e., RR = 1.0), was 3.78 (2.78–5.15). When considering the 1144 participants with household contact as the reference group, the crude RR of contact in the dormitory was 2.18 (1.57–3.03).

All the 33 RT-PCR test-positive cases after contact in the dormitory occurred in the largest cluster outbreak in Sendai City in 2020, which took place in dormitory A (School #1). To identify the factors creating the discrepancy in transmission risks between different dormitories, we surveyed and compared the infrastructural and lifestyle differences between the dormitories A and B, summarized in Table 3. Among the observed differences between the two, the residents lived in private living rooms in dormitory B, whereas two residents shared each living room in dormitory A. Another notable difference was that most of the residents in dormitory B were wearing masks when contacting the infected residents, whereas none of those in dormitory A were wearing masks when the outbreak occurred there.

**Table 3:** Facility features and infection control measures in two dormitories with different SARS-CoV-2 test positive rates.

	Dormitory A (School #1)	Dormitory B (School #2)
Male: female (n)	57 : 0	30 : 15
Age*	24 (23–25) years	27 (24–31) years
SARS-CoV-2 test positive, n (%)	33 (57.9%)	0 (0.0%)
Estimated number of index cases who first brought the infection to the dormitory (n)	3–7 primary cases who attended a large national traditional festival with meals and acquired COVID-19	2 primary cases ^†^ who had meals with a patient with COVID-19
**Facility features in each dormitory**		
Number of residents in each private living room	2 residents per room	Private room
Bathroom, toilet, and kitchen	Common use	Common use
Meals	Not served	Not served
Remove outdoor shoes in the dormitory	No^‡^	Yes^§^
Entry of non-residents to the dormitory	Not forbidden	Not forbidden
**Infection control measures in the dormitories at the time of screening test**		
Wearing masks in each private room	Not performed	Not performed
Wearing masks in shared space	Not performed	Performed by 50–70% of residents
Disinfection of commonly touched surfaces	Not performed	Performed everyday
Location of alcohol disinfection pumps	At the entrance	On each floor and in each shared space

*Median and interquartile range (25–75 percentile).

^†^These two patients noticed dysosmia several days after having meals with a COVID-19 patient and were later tested positive with SARS-CoV-2 in a medical facility different from our testing center. Both stayed in the dormitory for 2–3 days after the manifestation of their symptoms.

^‡^Shoe boxes are present in each private living room. Most residents remained barefoot in the living room. ^§^The shoe box is placed at the entrance of the dormitory. About half of the residents further changed their slippers when they entered the private living room.

### Comparison between dormitory-living and home-living students

As shown in the above sections, the COVID-19 transmission risk in dormitories may largely vary based on the infrastructural features and infection control measures adopted in each dormitory. Thus, to clarify the pure risk of living in group environments, we compared the transmission rate after contact with patients between the dormitory-living students and the home-living students in School #1. When the largest cluster outbreak occurred at School #1, 57 dormitory residents (1 with close contact, 56 with lower-risk contact) and 212 home residents (180 with close contact, 32 with lower-risk contact) contacted one or more COVID-19 patients. This cluster originated from a large-scale festival outside the school campus, during which the students contacted a COVID-19 patient by having meals together and sharing dishes. SARS-CoV-2 test positivity was confirmed in 33 (57.9%) of the 57 dormitory-living students and in 20 (9.4%) of the 212 home-living students. The rate was significantly higher among the dormitory-living students (φ = 0.498, *p* < 0.0001). When using the home-living students in contact with patients as the reference group, the calculated crude RR (95% CI) of acquiring the infection among dormitory-living students was 6.14 (3.83–9.84).

### Relationship between local epidemics and household transmissibility

**Figure 5:**
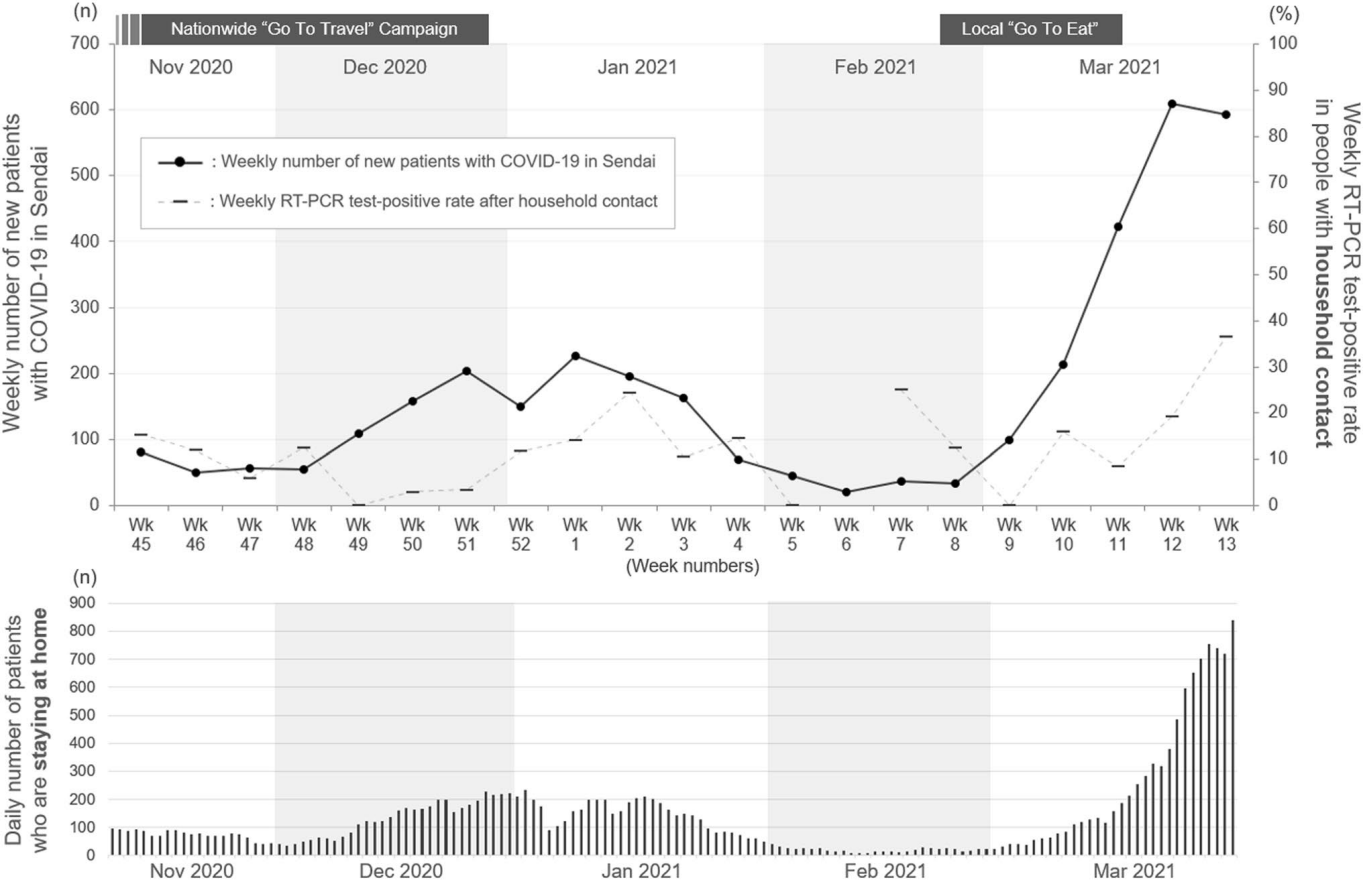
Change in local epidemic status and transmissibility of COVID-19 following household contacts. The black solid line represents the weekly number of newly diagnosed cases of COVID-19 in Sendai City. The gray broken line represents the weekly RT-PCR test-positive rate in people with a household contact who were tested at the drive-through COVID-19 testing center. The change in COVID-19 transmissibility after household contact appeared to be delayed by several weeks from the changes in local epidemic status. The lower half of the table shows the chronological change in the daily total number of infectious patients staying at their homes in Miyagi Prefecture (i.e., the prefecture where Sendai City is located).

Finally, to investigate the seasonality in the transmission of COVID-19 in a group living environment, the change in the weekly number of new patients in Sendai City and weekly positive RT-PCR test rate after household contact between November 2020 and March 2021 were evaluated. The changes in these two factors during the study period are shown in Fig. 5. The change in COVID-19 transmissibility after household contact did not precede changes in local epidemic status. Rather, the change in household transmissibility appeared to occur several weeks after the changes in local epidemics. The lower half of the table shows the chronological changes in the daily total number of COVID-19 patients in the infectious period who quarantined themselves at their homes without being admitted to hospitals or quarantine hotels. This line graph is almost synchronized with the chronological changes in household COVID-19 transmissibility.

**Figure 6:**
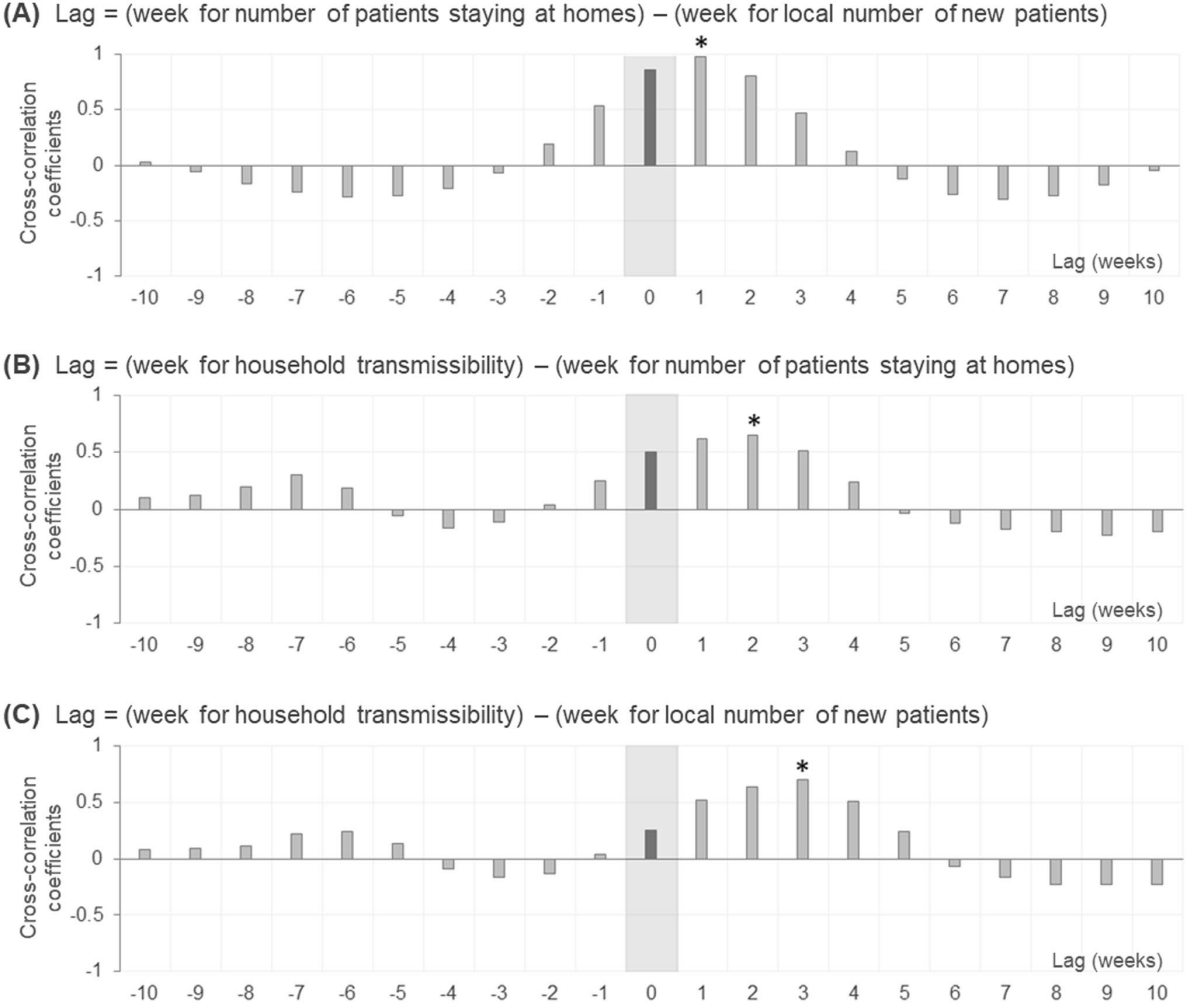
Cross-correlograms between local epidemics, number of patients staying at home, and household COVID-19 transmissibility. The local COVID-19 epidemic status is represented by the weekly number of newly diagnosed COVID-19 patients. The gray filled areas show the cross-correlations between two simultaneous variables with no time lag. The asterisk indicates the lag that produces the largest cross-correlation coefficient, suggesting a time delay between the two assessed variables.

To evaluate the significance of the delayed effect of local epidemics and number of standby patients staying at their homes on subsequent household COVID-19 transmissibility several weeks later, a time delay analysis was performed and cross-correlograms with the pairs of these variables were built (Fig. 6). The delay between the local epidemic status (i.e., the number of new patients) and number of standby patients staying at their homes was 1 week, and that between the number of standby patients staying at their homes and household transmissibility was 2 weeks. The delay between the local epidemic status and household transmissibility was the sum of these two delays, i.e., 3 weeks. The local government (Miyagi Prefecture) announced the daily number of potentially infectious patients staying at their homes.

## Discussion

This study examined and evaluated the risks of group living environments, such as dormitories or households, for acquiring the COVID-19 infection by using RT-PCR test results from 4550 participants (including 355 RT-PCR tested positive cases) with a history of recent contact with patients. The results showed that the risk of secondary transmission, measured by the SARS-CoV-2 RT-PCR test positivity rate after recent contact with patients, was highest among the 120 participants with contact in dormitories (27.5%), and was similar for the 1144 individuals with household contact (12.6%) and the 1034 with close contact outside the residences (11.3%). The transmission rates in these three groups were significantly higher than those of the 2252 participants with lower-risk contact outside the residences (2.7%). Although the secondary transmission rate was significantly higher in dormitories, the transmission rates were much different between the three dormitories where contact occurred (57.9%, 0.0%, and 0.0%, respectively). In the dormitory where the largest cluster outbreak occurred, none of the residents were wearing masks inside, and two residents were sharing living rooms during the outbreak. Despite the small number of evaluated dormitories, this fact may imply that the risk of secondary transmission after contact in dormitories would be largely affected by lifestyle and infection control measures among residents. Regular and sufficient implementation of appropriate additional infection control measures in dormitories, such as wearing masks outside the living rooms, performing effective room ventilation, disinfecting the commonly-touched surfaces, maintaining hand hygiene, physical distancing, and avoiding gathering with large numbers of residents, are required to suppress the secondary transmission risks during pandemics^11,17^.

Another notable finding of this study is that household secondary transmission was the main form of COVID-19 transmission among non-adults aged < 18 years. As shown at the bottom of Table 1, the rate of household secondary transmission was much higher than that after close contact outside homes (mostly at schools) among the non-adults. In addition to the infection control measures at schools, each non-adult parent should also regularly perform infection prevention measures and precautions to prevent bringing the infection home and exposing their children. Several previous reports suggested that the risk of household secondary transmission was higher in adults than in non-adults^18–21^, which was not observed in our study. This research displayed similar risk of household secondary transmission in adults and non-adults. The reasons for this discrepancy are unclear, but a possible theory suggests different rates of spousal contact during a pandemic between different countries and ethnicities^14^. Further research is needed to confirm whether the risk of household secondary transmission differs between adult and non-adult family members.

The exact mechanism underlying the elevated risk of secondary transmission in group living environments is currently unclear. Increased opportunities for close physical contact and a low rate of wearing masks in group living environments, both of which increase the risk of droplet infection, are among the primary conceivable reasons. In addition, the possibility of contact infection by fomites via high-touch surfaces or shared meals might be also higher in group living environments. Many transmissible diseases, including SARS-CoV-2, have been reported to spread not only by droplet or aerosol transmission but also by contact infection, in which the pathogens are transmitted via food or commonly-touched surfaces^22–24^. Theoretically, group living and sharing equipment with others certainly increases the risk of contact transmission. The managers and residents of group living facilities, including dormitories and elderly group living homes, should take additional countermeasures, such as cleaning and disinfecting high-touch surfaces or performing proper hand hygiene, to suppress not only the risk of droplet infection but also fomite infections.

As shown in Fig. 5, chronological changes in household COVID-19 transmissibility appeared to occur several weeks after changes in local epidemic status. One of the possible theories may be an increase in the number of patients who cannot be admitted to hospitals or quarantine hotels based on the strained receiving capacity in the locality several weeks after the occurrence of a local outbreak and are standing by in their homes. Although the exact relationship between the household COVID-19 transmissibility and local epidemics is uncertain, the results suggest that changes in household transmissibility may not be among the major driving factors that regulate local epidemic status. Conversely, household transmissibility may be affected by local epidemic status, possibly based on the local upheaval of hospitals and quarantine hotels, which would result in a longer household contact with a family member with COVID-19. Based on the line graphs in the figure, a possible explanation may be an increase in the duration of household contact with infectious family members with COVID-19, resulting from the local upheaval of receiving capacity in the hospitals and quarantine hotels, could increase the household secondary transmission rate. These findings suggest that quarantining themselves at their homes with other family members without being admitted to hospitals or quarantine hotels would be a significant risk for transmitting COVID-19 with whom they live.

One limitation of this study was that we did not evaluate whether the recent contact with a COVID-19 patient was before or after the clinical onset of the patient. Since the infection risk may be different before and after the appearance of clinical symptoms, or by the presence of symptoms^25,26^, collecting data about the clinical symptoms of the patient at the time of contact would be desired in similar research as a possible predisposing risk in addition to the presence of close contact history. Another limitation was that only two dormitories were evaluated in detail regarding the infrastructural features and lifestyle of the residents. Since the transmission rate of the COVID-19 infection in dormitories is largely affected by the structure, lifestyle, and infection control measures in each dormitory, calculating the exact relative risk of acquiring the infection in those conditions is practically difficult. What our results clearly show is that group living environments are an independent significant risk from close contact history for acquiring the infection, if appropriate countermeasures are not sufficiently implemented. Because dormitory-living students may have greater opportunity and extended length of exposure to environmental hazards than home-living students, additional infection control measures are required.

In conclusion, contact with patients with COVID-19 in group living environments, such as dormitories or households, is a significant independent risk factor for acquiring the disease. Household secondary transmission is suggested to be the current main form of infection among non-adults and is synchronized to the local epidemic status with changed local capacity for quarantining infectious residents, such as in hospitals or quarantine hotels. To suppress the secondary transmission risk in group living environments, appropriate infection prevention measures, such as physical distancing, wearing masks, effective ventilation, and quarantining infectious residents, are needed to suppress the risk of secondary transmission among group living residents.

## Methods

### Eligibility criteria

Individuals who underwent RT-PCR testing at our nasopharyngeal swab testing center were contacted in advance by the local public health centers based on contact tracing, or those who spontaneously made phone calls to the public health centers based on their contact history and/or symptoms suggesting a COVID-19 infection. The participants had been assessed for their level of contact with a COVID-19 patient and their need to undergo RT-PCR test was evaluated by the official health centers. All registered individuals with recent contact history were advised by the staff of local public health centers on the first phone call to self-quarantine until the RT-PCR test returned negative results. Among the subjects tested during the 9-month study period (July 2020 to March 2021), those asked for their recent contact history and with available data concerning the contact level (close/lower risk) and place (household/group living/outside the residences) were considered eligible for analysis. Tested individuals with no recent contact history with COVID-19 patients, together with those with unidentified contact places were excluded from this study. The study period included the campaign periods of the nationwide “Go To (Domestic) Travel” campaign from July 2020 to December 2020 and the local “Go To Eat” campaign from February 2021 to March 2021. The former campaign was a central government policy to financially support domestic travel for travelers with the purpose of supporting the tourist industry damaged by the pandemic, and the latter was a local government policy to financially support eating-out expenses for customers with the purpose of supporting the damaged local eating-out industry. Both campaigns were suspended following nationwide or local COVID-19 outbreaks. During the study period, the rate of going out widely changed among citizens and the area experienced irregular COVID-19 outbreak; however, the criteria for RT-PCR screening tests and contact closeness did not change. The study period was well before the replacement of major viral strains spreading in the locality from the original strains to N501Y mutant strains in May 2021.

### Definition of close contact history

In Japan, close contact was defined by the fulfillment of all following four criteria during the study period: 1) contact with a COVID-19 patient between 2 days before and 14 days after the onset of symptoms, 2) no usage of masks, 3) distance less than 1 m, and 4) more than 15 min of contact^27–29^. Concerning the contact with asymptomatic patients with COVID-19, contact from 2 days before the patient underwent a diagnostic test with positive result was applied as the first criterion. The ways of contact included physical contact (e.g., nursing, caregiving, sports activity, playing at schools, bathing, kissing, or sexual intercourse), conversation, singing, having a meal, and others. Direct exposure to the contaminated body fluid from patients (saliva, respiratory secretion) without proper personal protective equipment were also regarded as close contact. Lower-risk contact was defined as being in the same place as the COVID-19 patients, but without fulfilling the above-described criteria for close contact.

### Real-time RT-PCR

Nasopharyngeal swab tests were performed in all participants. SARS-CoV-2 viral RNA positivity was checked using real-time RT-PCR analyses for detecting SARS-CoV-2 nucleocapsid protein set no.2 (N2) gene^30^. The primer/probe set designed by the National Institute of Infectious Diseases in Japan (forward primer: NIID_2019-nCOV_N_F2; reverse primer: NIID_2019-nCOV_N_R2; TaqMan probe: NIID_2019-nCOV_N_P2) was used^31^. The reaction mixture comprised 4 × TaqMan Fast Virus 1-Step Master Mix (Thermo Fisher Scientific, Waltham, MA, USA), the aforementioned primer/probe set, and nuclease-free water. Thermal cycling was performed as reported in a previous research^16^.

### Detail of the dormitory environments

This study included 120 dormitory residents from three dormitories who contacted other infectious residents. The first dormitory (dormitory A) had 57 contacted residents tested at a drive-through testing center, the second dormitory (dormitory B) had 45 contacted residents, and the last dormitory (dormitory C) had 18 contacted residents. Dormitory A was the place where the largest cluster of COVID-19 in the city occurred in 2020. To identify factors behind the different secondary transmission rates of COVID-19 among dormitories, we further investigated infrastructural features, demographics and lifestyles of the residents, and infection control measures implemented at the occurrence of COVID-19 in dormitories A and B. The collected data were as follows: age and sex of the residents; estimated number of primary cases with COVID-19 who first brought the infection into dormitories; number of residents living in each private living space; presence of meal service; situation of footwear in the living space; sharing status of bathroom, toilet, or kitchen; and implemented infection control measures upon the occurrence of COVID-19 among residents. Because dormitory C had a relatively small number of contacted residents, these detailed data were not collected from this dormitory.

### Statistical analysis

The distribution of each quantitative variable is described as median and interquartile ranges (25–75 percentiles) due to the non-normal distributions in most evaluated variables. The prevalence of categorical variables between the two groups was compared using the chi-square test. The effect size for the chi-square test was reported with *φ*. The SARS-CoV-2 RT-PCR test positivity rates were compared between the groups with different places of contact, and further evaluated after dividing the individuals into adults aged ≥ 18 years and non-adults aged < 18 years. To further verify the risk of contact with patients in group living environment by multivariate analyses, binary logistic regression analyses were performed using the SARS-CoV-2 RT-PCR test result as the response variable. The explanatory variables included demographic or contact-related information of interest, as well as additional variables that were suggested to be significant predictors of RT-PCR test positivity in the univariate analysis. To calculate the crude RR and its 95% CI of RT-PCR test positivity in each group with different group living environments (household/dormitory), participants with close contact outside the residences (i.e., workplace, school, or eating place) were used as the control group to determine the test positivity rate. For the time delay analyses, the cross-correlation coefficient between local epidemics, local capacity for quarantining infectious patients, and household transmission rates for time lag between − 10 and + 10 weeks were calculated. The data regarding the local epidemic status and the number of potentially infectious patients staying at their homes were announced by the local governments (Sendai City and Miyagi Prefecture). Statistical significance was set at *p* < 0.05. Statistical analyses were performed using IBM SPSS Statistics (version 22.0, IBM, USA) and R Statistical Software (version 4.0.5, R Foundation, Austria).

### Ethical statement

This study was approved by the Institutional Review Board of the Tohoku University Graduate School of Medicine (approval number: 2020–1-847). All procedures were performed in accordance with the current version of the Declaration of Helsinki, as revised in 2013. The Institutional Review Board waived the need for written informed consent from participants due to the urgent need to collect necessary data about the emerging infectious disease without increasing the risk of its spread. The process of informed consent was thus secured in an opt-out manner.
